# Quantifying the Quality of Web-Based Health Information on Student Health Center Websites Using a Software Tool: Design and Development Study

**DOI:** 10.2196/32360

**Published:** 2022-02-02

**Authors:** Anagha Kulkarni, Mike Wong, Tejasvi Belsare, Risha Shah, Diana Yu Yu, Bera Coskun, Carrie Holschuh, Venoo Kakar, Sepideh Modrek, Anastasia Smirnova

**Affiliations:** 1 Department of Computer Science San Francisco State University San Francisco, CA United States; 2 School of Nursing San Francisco State University San Francisco, CA United States; 3 Department of Economics San Francisco State University San Francisco, CA United States; 4 Department of English Language and Literature San Francisco State University San Francisco, CA United States

**Keywords:** online health information quality, information quality metrics, automated quantification tool, student health center websites, digital health, health information, health information websites, adolescents, online health, infodemiology, public health, health websites

## Abstract

**Background:**

The internet has become a major source of health information, especially for adolescents and young adults. Unfortunately, inaccurate, incomplete, or outdated health information is widespread on the web. Often adolescents and young adults turn to authoritative websites such as the student health center (SHC) website of the university they attend to obtain reliable health information. Although most on-campus SHC clinics comply with the American College Health Association standards, their websites are not subject to any standards or code of conduct. In the absence of quality standards or guidelines, monitoring and compliance processes do not exist for SHC websites. Thus, there is no oversight of the health information published on SHC websites by any central governing body.

**Objective:**

The aim of this study is to develop, describe, and validate an open-source software that can effectively and efficiently assess the quality of health information on SHC websites in the United States.

**Methods:**

Our cross-functional team designed and developed an open-source software, QMOHI (Quantitative Measures of Online Health Information), that assesses information quality for a specified health topic from all SHC websites belonging to a predetermined list of universities. The tool was designed to compute 8 different quality metrics that quantify various aspects of information quality based on the retrieved text. We conducted and reported results from 3 experiments that assessed the QMOHI tool in terms of its scalability, generalizability in health topics, and robustness to changes in universities’ website structure.

**Results:**

Empirical evaluation has shown the QMOHI tool to be highly scalable and substantially more efficient than manually assessing web-based information quality. The tool’s runtime was dominated by network-related tasks (98%), whereas the metric computations take <2 seconds. QMOHI demonstrated topical versatility, evaluating SHC website information quality for four disparate and broad health topics (COVID, cancer, long-acting reversible contraceptives, and condoms) and two narrowly focused topics (hormonal intrauterine device and copper intrauterine device). The tool exhibited robustness, correctly measuring information quality despite changes in SHC website structure. QMOHI can support longitudinal studies by being robust to such website changes.

**Conclusions:**

QMOHI allows public health researchers and practitioners to conduct large-scale studies of SHC websites that were previously too time- and cost-intensive. The capability to generalize broadly or focus narrowly allows a wide range of applications of QMOHI, allowing researchers to study both mainstream and underexplored health topics. QMOHI’s ability to robustly analyze SHC websites periodically promotes longitudinal investigations and allows QMOHI to be used as a monitoring tool. QMOHI serves as a launching pad for our future work that aims to develop a broadly applicable public health tool for web-based health information studies with potential applications far beyond SHC websites.

## Introduction

### Background

Since the early 1990s, internet has been a major source of health information, and its adoption among health care providers and patients has been growing ever since [1-3]. Health information provided on various internet sites often varies greatly in terms of the quality and reliability of the content [1,4-7]. Common assessments include that the information is too technical or difficult to read, the website is difficult to use (ie, search or navigate), or is unreliable. Assessment instruments have been proposed to help users navigate the high variability in the quality of web-based health information [8-14]. For instance, DISCERN uses questionnaires to help users assess the quality of health information [9]. Similarly, guides for publishing quality health care information have been proposed [10,15,16]. However, the assessment instruments have to be applied manually, typically by field experts, and the implementation of guidelines is not enforced [17,18]. Therefore, the adoption and implementation of the proposed best practices for web-based health information have been limited and nonsystematic.

Adolescents and young adults are particularly vulnerable to the risks arising from inaccurate, incomplete, or outdated web-based health information because they tend to rely heavily on the internet for their information needs [19-24]. Studies have found that adolescents and young adults are savvy internet users who are aware of the problems with the quality of web-based information and thus prefer to use authoritative websites for health information [22]. In a qualitative study with focus groups, usability tests, and in-depth interviews, participants preferred institutional sources of health information over private websites [25]. One such prominent institutional source of health information is the student health center (SHC) websites at higher education institutes (HEIs).

In 2016, approximately 41% of the students aged 18-24 years were enrolled in an HEI with a higher proportion of female attendees than male attendees (43% female attendees vs 38% male attendees) and growing racial and ethnic diversity of the student population, as reported by the National Center for Education Statistics [26]. On the basis of a national study of universities and their SHCs, 85% of the 214 participating higher education institutions in the United States had an SHC website and on-campus clinic in 2015 [27]. SHC websites are commonly perceived as an extension of the SHC clinics and thus are regarded as an authoritative and credible source of health information by adolescents and young adults [27-29]. Rather than physically visiting an SHC clinic on a university campus, most students now make their first contact with an SHC through their website. As such, SHC websites are a leading accessible source of high-quality health information for adolescents and young adults in the United States.

Most on-campus SHC clinics that students visit in person comply with the American College Health Association (ACHA) standards [30]. More than 800 HEIs in the United States have ACHA membership, which provides a healthy campus framework, health and wellness consulting, patient satisfaction assessment service, and national college health assessment to improve overall health status on campus. However, ACHA is limited to on-campus SHC clinics and does not extend its services to SHC websites. As a result, the quality of health information on SHC websites is not monitored by any central governing body.

### Objectives

On the basis of these observations, this study aims to develop, describe, and validate an open-source software that can effectively and efficiently assess the quality of health information on SHC websites in the United States. The tool QMOHI (Quantitative Measures of Online Health Information) provides a suite of quantitative measures of information quality, which can be used by health care administrators, researchers, and practitioners to assess, monitor, and improve the information posted on SHC websites.

## Methods

### QMOHI System Design and Implementation

#### Overview

A cross-functional team consisting of computer scientists, a software developer, a public health researcher, a nurse practitioner, an economist, and a linguist outlined the framework and capabilities necessary to assess information quality on SHC websites. The team identified exemplars of high-quality SHC websites and then worked with subject matter experts to identify distinct attributes of web-based information that modeled quality, such as topic coverage, accessibility, navigation, readability, sentimentality, and polarity. The team iteratively refined the initial framework and incorporated key measures of quality into the QMOHI software tool. For the development of the QMOHI software, the Agile methodology was adopted to facilitate iterative design, development, testing, and feedback workflow [31]. We evaluated the QMOHI for the following key properties:

- Scalable—ability to provide quality assessment for a large number of SHC websites efficiently
- Generalizable—ability to conduct a quality assessment of any topic of information on SHC websites
- Robust—ability to be redeployed periodically on SHC websites while adapting to changes in website content and structure
- Fully automated—ability to perform the quality analysis without any human intervention

The QMOHI tool was designed with the assumption that the user would specify two key pieces of input information: (1) the list of universities of interest and (2) the topics of interest, in the form of keywords. These user inputs guided the information gathering and analysis conducted by QMOHI. At a high level, the QMOHI tool was organized into three key phases: phase 1—locate SHC website, phase 2—gather the related information and specific text on the topic of interest from the SHC website, and phase 3—assess the quality of information.

**Figure 1:**
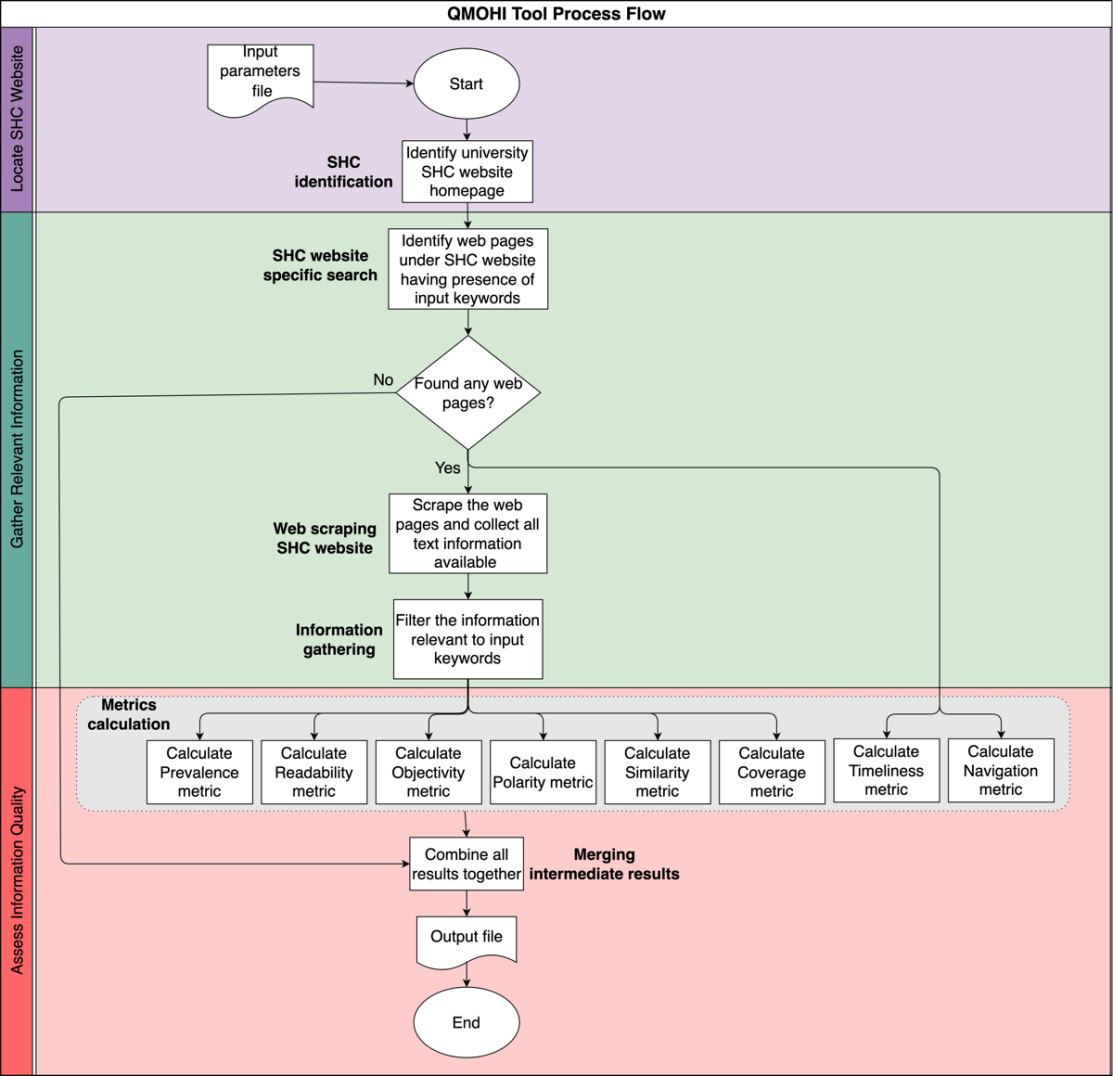
Process flow of the QMOHI (Quantitative Measures of Online Health Information) tool. SHC: student health center.

Figure 1 provides a flowchart for the QMOHI tool, in which the 3 phases are delineated using different background colors.

#### Phase 1: Locate SHC Website

QMOHI first found the SHC website, more specifically, the web address (URL) of the SHC website for each of the universities specified by the user. We designed and developed an algorithmic approach for this task that consists of four simple steps:

1. Constructing a search query by joining the given university name with the phrase *student health center* (eg, *Texas A & M University Central Texas student health center*).
2. Running the search query using a commercial search engine (eg, Google Custom Search application programming interface).
3. Retrieving URL of the first result; if the URL was not from the *.edu* domain, the next URL was retrieved. This was repeated until the third URL was processed. If none of the top 3 URLs were from *.edu* domain, it was then concluded that the SHC website could not be found for this university and ended.
4. Sanitizing the retrieved URLs to obtain the definitive URL for the SHC home page by checking whether the URL redirected another URL with the help of the Selenium WebDriver; if yes, then the new URL was used, and the sub-URLs such as*/contacts*, */appointments*, and */location* from the URL were removed.

This multistep approach was necessary because of the large variability found in the web addresses of SHC websites. There are no standards or even commonly accepted conventions for SHC website naming or hosting structures. The following 6 California State universities illustrate the scope of variability among sister HEIs. All six California State universities mentioned here have a different approach for formulating their SHC web address:

1. California Polytechnic State University, San Luis Obispo: hcs.calpoly.edu
2. California State University, Bakersfield: www.csub.edu /healthcenter
3. California State University, Stanislaus: www.csustan.edu /health-center
4. California State University, San Bernardino: www.csusb.edu/student-health-center
5. California State Polytechnic University, Pomona: www.cpp.edu/~health
6. San Francisco State University (SFSU): health.sfsu.edu/

#### Phase 2: Gather Topical Information

The core task of this phase was to download all the textual information related to the topics of interest from the SHC website identified in the previous phase. To operationalize this process, we used the following approach:

1. Constructing a disjunctive search query from all the topic keywords specified by the user. (Example query: *Corona, coronavirus, COVID; site: health.sfsu.edu*)
2. Using a commercial search engine (eg, Google Custom Search application programming interface) to conduct a site-specific search with the above query against the SHC website. (Site-specific search returns only those webpages that are hosted under the specified site, in our case, the SHC website.)
3. Downloading all webpages in the search result. In addition, the URLs of these webpages were saved. The URLs would be required to compute one of the quality metrics in phase 3.
4. From each webpage, every sentence containing any of the input keywords (anchor sentences) and 5 sentences before and after it (context sentences) were extracted. This step filtered out nonrelevant content by anchoring and localizing the extraction process around the topic keywords.
5. Consolidating all the information extracted in step 4 from all the result webpages of SHC. The duplicate sentences from the consolidated information were filtered.

The data gathered by this approach formed the basis for the analysis conducted in the next phase.

#### Phase 3: Quantify Information Quality

##### Overview

The QMOHI tool computed an array of quantitative measures of quality for the gathered information in this phase. Eight quality metrics—readability, coverage, prevalence, objectivity, polarity, navigation, timeliness, and similarity—were implemented in QMOHI. Each quality metric captured a unique aspect of web-based information that was important in the context of health care information dissemination and reflected the multidimensional nature of information quality [32,33]. Every metric was designed and developed such that its computation was completely automated to facilitate large-scale studies. These metrics and the motivations behind them are described as follows.

##### Metric 1: Readability (Reading Level)

If the information provided on an SHC website used a simple, easy-to-understand language, then it was more likely to be understood and correctly applied. In contrast, if the information on the SHC website used specialized medical terminology, then an average college student would be unlikely to find it accessible. There is an extensive body of research in the context of physician–patient communication that transfers over to web-based health information communication [34-37]. We referred to this concept as *information understandability* and quantified it using the Flesch–Kincaid readability tests [38]. The Flesch–Kincaid readability tests consist of two metrics: Flesch Reading Ease (FRE) and Flesch–Kincaid Grade Level (FKGL), which use linguistic properties of the textual content to score its readability, as follows:

1. Counting the number of syllables, words, and sentences for the consolidated content gathered in the previous phase
2. Computing the FRE metric: 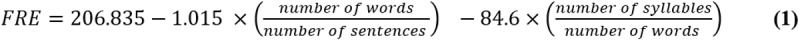
3. Computing the FKGL metric: 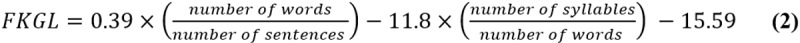

A higher score for the FRE metric indicated that the text is easy to read, and a lower score indicated that the material is difficult to read. The scores computed by the FKGL metric corresponded to US grade levels. We applied these metrics to assess the understandability of the information provided on SHC websites.

##### Metric 2: Prevalence

The volume of relevant information was a crucial aspect of information quality [39]. When relevant information was mentioned in passing and never repeated, it was likely to be overlooked or misunderstood [40]. Therefore, the quantity of relevant information provided on SHC websites was also important. One SHC website may provide just a sentence about the topic of interest, whereas the other may include a detailed post, along with additional reading pointers. The prevalence metric captured this intuition by computing the cumulative frequency of all input keywords present in the information gathered from the SHC website:



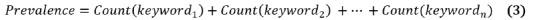



##### Metric 3: Coverage

Some health care topics required several keywords to be completely expressed. If an SHC website contained more of these keywords, then it provided more in-depth and complete information about the given health topic. Here, we defined our next metric, coverage, to model this intuition as the ratio of the number of keywords found on the SHC website to the total number of input keywords:



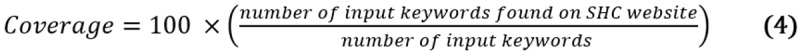



The coverage metric ranged from 0 to 100 based on the number of input keywords found, where 0% indicated that none of the input keywords were found on the SHC website and 100% indicated the presence of all input keywords on the SHC website. Although the coverage metric can provide the percentage overlap between input keywords and information on SHC, this metric alone should not be considered as completeness of the information on the health topic. This is because input keywords might be only a subset of all keywords related to a particular health topic. As such, the utility of both prevalence and coverage metrics depended on the comprehensiveness of the input keywords for a specific health topic.

##### Metric 4: Sentiment—Objectivity

High-quality health information is high in factual information content and low in unsupported opinions. A measure of these 2 directly opposing qualities can be expressed as *objectivity* and *subjectivity*, respectively. Objectivity is an information quality metric that quantifies the extent to which information is impartial [40]. TextBlob [41] provided sentiment analysis, including subjectivity scoring algorithms based on a curated weighted lexicon approach. The subjectivity scores were bounded in 0 and 1, where 1 is the most subjective and 0 is the most objective. In this work, we computed the subjectivity score of the information gathered in the previous phase of QMOHI using TextBlob, and then defined the objectivity metric as 1 (*subjectivity*).

##### Metric 5: Sentiment—Polarity

Along with the objectivity measure, polarity is important for assessing the quality of the information on the SHC website. The same information about the evidence on health effects can be framed either positively or negatively [42], for example, “This disease can be difficult to cure entirely if detected in later stages” and “This disease can be easy to cure entirely if detected in early stages.” Both sentences express similar meanings, but their polarities are contrary. Critically, different positive and negative framing can shift people’s preferences, even if the options are objectively equivalent [43]. Polarity of the health information on the SHC website may affect people’s decisions about health services.

The polarity metric quantified the positivity, negativity, or neutrality of the health information on the SHC website. For this tool, the polarity score was computed using TextBlob’s sentiment analysis feature [41] on the health information collected from the SHC website. This score ranged between –1 and 1, where 1 indicated a strongly positive statement, –1 indicated a strongly negative statement, and 0 indicated a neutral statement, for example,

1. “They have the best available doctors, equipment and treatment facilities.” This sentence shows affirmation. It has a polarity score of 0.7.
2. “If the cancer is located only in the breast, the 5-year relative survival rate of people with breast cancer is 99%.” This sentence is neutral; it has a polarity score of 0.
3. “The service of health center AAA is atrocious for XYZ.” This sentence shows negative expressions, it has a polarity score of –0.39.

##### Metric 6: Navigation (Number of Clicks)

Well-designed websites make it easy for users to find the information they need, minimizing the demand for users’ time and effort. This intuition was modeled by the navigation metric that computed the minimum number of clicks needed to reach the desired content when starting from the SHC home page. At a high level, the algorithm for computing this metric was designed to simulate the website traversal path a human would follow when looking for specific information on SHC websites. To find the content closest to the SHC home page (minimum number of clicks), this exploration was conducted in a breadth-first search. To operationalize this logic, a customized tree data structure with a queue was used to prioritize the webpages (URLs) that had to be checked iteratively.

As shown in Textbox 1, the expected input by the navigation algorithm is the SHC home page URL and the URLs for webpages retrieved by site-specific search in phase 2. The first node to be created in the tree data structure was for the SHC home page (line 1) and was added to the queue (line 2). At each iteration, a node at the head of the queue was obtained (line 4). The program was terminated if the level of the current node was >10 (line 5).

It was assumed that keywords’ content was not present on this SHC website, and a special value of –1 and empty trace was returned for the navigation metric to indicate the same (line 6). If the current node’s URL matched with any of the target webpage URLs (line 8), then the program ended by returning the level (number of clicks) and trace of the current node (line 9). When a current node’s URL did not match any of the target URLs, all hyperlinks on the current page were extracted (line 11). The hyperlinks that were external to the SHC web domain were filtered out. For the remaining hyperlinks, a new tree node was created that was attached to the current node as a child node (lines 13-19). This process was repeated until the queue was empty (line 3) or until either of the other 2 stopping criteria were met (lines 6 or 9).

**Algorithm for the navigation metric.**

Input: (1) SHC home page URL and (2) Target pages: URLs for webpages retrieved by site-specific search in phase 2

Output: (1) Minimum number of clicks (Navigation metric) and (2) Trace (An ordered list of URLs—path from SHC home page to closest target page)

1. Initialize: Tree data structure with one node (root) containing:
  URL: SHC home page URL
  level: 0
  trace: SHC home page URL
2. Add root node to the queue
3. while queue is not empty do
4. Pop the node at the head of queue
5. if level of current node >10
6. return –1 and empty trace
7. end if
8. if current node’s URL matches with any of the target page URLs
9. return level and trace of the current node
10. else
11. Extract all the hyperlinks from the contents of the node’s URL
12. Filter out the hyperlinks that are outside of SHC web domain
13. For each hyperlink *h*
14. Create a new child node where:
15. URL: *h*,
16. level: parentNode.level+1,
17. trace: append ( *h* to parentNode.trace)
18. Add the new node to the queue
19. End for
20. end if
21. end while

##### Metric 7: Timeliness

Health care information is dynamic in which new or improved treatments are brought to the market, sometimes replacing existing treatments, or relegating them to be used only under specific conditions. SHC websites should be regularly checked and revised to stay current with the latest health information, removing deprecated information, and reorganizing existing information to reflect critical health care priorities, such as vaccine availability during a pandemic. Outdated information, without the advice of a trained health care provider, can lead to suboptimal decisions. Therefore, the timeliness of information is an important aspect of information quality. Webpages on a particular university’s SHC website may be modified at different times, and certain webpages may be updated more often than others. It is important to know when the information was last updated on the SHC webpage from which the information of a certain health topic is referred.

The timeliness metric quantified this insight through the *last revised timestamps* on SHC webpages that contain the input keywords. These timestamps were fetched from the webpage headers with the *Last-Modified* tag, and if absent, they were marked as –1. For webpages with a –1 timeliness metric score, the recency of content could not be determined. With more recent timestamps, the probability of the latest information increased.

##### Metric 8: Relevancy or Similarity

Relevancy describes the extent to which information is applicable and helpful to users’ information needs [40]. Relevancy of health information on the SHC website is contextual and subjective; as such, it is difficult to assess directly. We can approximate relevancy by calculating the lexical similarity between the information on the SHC website and an *ideal* reference document, which is a document, manually created by experts, containing all the information relevant to the health topic of interest (perhaps using Centers for Disease Control and Prevention references, for example). To operationalize this intuition, we used a cosine similarity function, which is defined as follows:



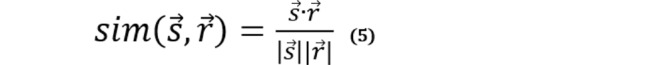



where 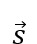 is a numeric vector representation of the collated SHC website content gathered in phase 2 and 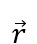 is a numeric vector representation of the ideal reference document. Similarity values closer to 1 indicated that the relevance of the topical information on the SHC website is high, whereas values closer to 0 implied low relevance.

### Experimental Setup

#### Overview

The following set of experiments provide an empirical evaluation of the QMOHI tool on three key performance metrics:

1. Scalability—measured by timed trials versus human annotators to navigate an SHC website with a specific information goal and performance benchmarking trails
2. Generalizability—evaluated by comparing results with varying information specificity and looking for poor performance
3. Robustness—evaluated by computing quality metrics over time as SHC websites change in both content and structure

#### Experiment 1: Scalability and Efficacy

##### Overview

The first experiment investigated the scalability of the QMOHI tool using two methods: (1) by comparing the time needed by human annotators to find topically relevant information on the SHC website to that by QMOHI and (2) through performance benchmarking of the QMOHI tool by measuring its end-to-end runtime and studying the breakdown of the runtime.

##### Method 1

For the first method, 200 universities were chosen at random from a larger set of all 4-year, public, bachelor’s granting universities in the United States (N=549). The list of 200 universities was shuffled and partitioned into 20 equal groups to allow for timing the task at the group level rather than at the university level to smooth out any individual university-level idiosyncrasies. Two annotators conducted the task on all 20 groups for the health topic of long-acting reversible contraceptives (LARC), which was represented by the following keywords: *IUD, intrauterine device, IUI, intrauterine implant, contraceptive implant, contraceptive shot, contraceptive injection, Depo Provera, and Depo-provera*. The annotators were instructed to perform the following steps:

1. Find the SHC website of the university with Google search.
2. Calculate the minimum number of clicks needed to reach the first mention of any of the given keywords from the SHC home page. The starting point is the SHC website’s home page, with the number of clicks as 0.
3. Indicate *No mention* if none of the keywords were found on the SHC website.
4. Record the time required to perform the whole task on every group of 10 universities.

The task of finding the first topically relevant webpage on the SHC website could be considered equivalent to computing the navigation metric using the QMOHI tool. Thus, the time required by QMOHI to compute the navigation metric was compared with the annotation time.

##### Method 2

The authors also conducted performance benchmarking for the QMOHI tool by measuring its end-to-end runtime. Specifically, 20 (10%) universities, selected at random from the subset of 200 universities known to have SHC websites, were searched using the QMOHI tool for 2 health topics (topic 1: *pap smear* and topic 2: *all contraception*) on cloud servers. The *pap smear* topic mapped to 6 keywords query, each a variation of *pap smear* and *pap test*. The *all contraception* topic was represented using 37 keywords, including the following: *birth control, contraceptive implant, hormonal IUD*, and others. The runtime of the tool for every university and every topic was recorded. In addition, the time spent by the tool gathering the information (network time) versus processing the information (compute time) was recorded to facilitate a thorough performance analysis.

Cloud servers provide accessible and reliable networks and dedicated infrastructure, as opposed to local student laptops or university infrastructure, which may be multipurpose or have unreliable networks. Aside from the operating system itself, the cloud server was set up to exclusively run QMOHI during benchmarking. The cloud server used was an Amazon EC2 *t2.large* instance, featuring dual central processing units with 8 GB memory, and the network throughput was profiled using *iperf* from an EC2 instance in Virginia to a local *iperf* server in California and measured an average 51.1 MiB/s over 3 connections. As the universities were all within the United States, transcontinental communications approached the upper bounds of network traversal.

#### Experiment 2: Generalizability

The second experiment examined the QMOHI tool’s ability to compute information quality metrics for a wide range of health topics. Specifically, the quality of information for the 4 health topics—COVID, cancer, LARC, and condoms—on the SHC website of SFSU was evaluated for this experiment.

The other part of this experiment tested QMOHI’s ability to work with narrowly focused health topics. The relevant information for such topics can be sparse and spread on SHC websites. Whether QMOHI can tackle these data challenges was examined by this experiment with the following two fine-grained health topics: hormonal intrauterine device (IUD) and copper IUD (Paragard), which are searched for on SHC website of the SFSU.

The set of input keywords used with QMOHI for each of the above health topics is given in the Multimedia Appendix 1.

#### Experiment 3: Robustness

Robustness is the ability of a software system to recover from errors, such as unexpected changes in input. Public health studies are often longitudinal, and data collection and analysis must be conducted periodically over a longer period of time. During this time, SHC websites might change both webpage content and structure (ie, file names, directories, and even complete URL changes). QMOHI can analyze website content regardless of changes in the website structure. To evaluate the ability of the QMOHI tool to extract content from moving targets, a longitudinal study was conducted on multiple health topics over a period of 3 months for a large set of universities. Specifically, the QMOHI tool was run on July 14, August 14, and September 14, 2020, on the SHC websites of 549 public universities in the United States for the following five health topics: pap smear, condoms, LARC, all forms of contraception, and superset of all above keywords.

Universities, topics, and keywords were kept consistent in all 3 executions of the QMOHI tool over 3 months.

## Results

### Experiment 1: Scalability

#### Method 1

**Figure 2:**
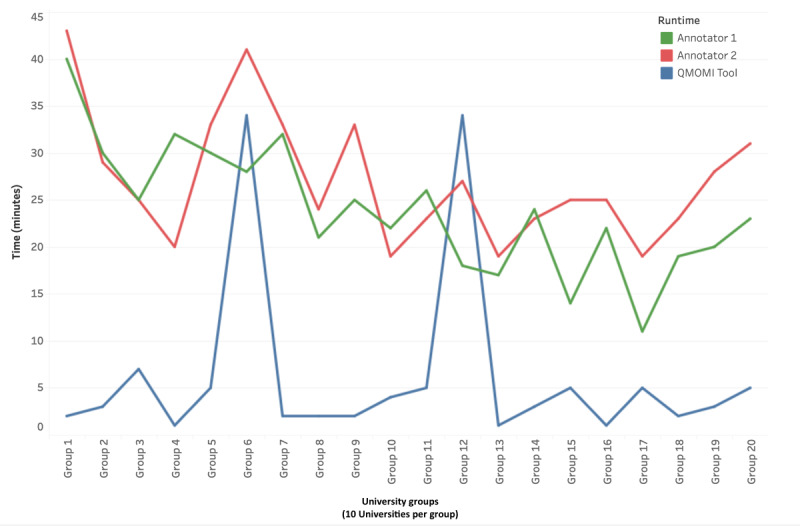
Scalability experiment: Runtime comparison chart for navigation metric. Group-level task times, with 10 universities per group. QMOHI: Quantitative Measures of Online Health Information.

The results for method 1 (navigation task) are listed in Table 1. The task was to find the first topically relevant information webpage on the SHC website. In the fastest scenario, QMOHI completed the task for 10 universities in 1 minute, which is an order of magnitude faster than the manual approach. However, the small difference between the maximum task times for the 2 approaches (approximately 41 minutes vs 34 minutes) was puzzling. To understand the underlying reason, Figure 2 provides zoomed-in data: group-level task times for each of the 20 groups. These data reveal two outliers: groups 6 and 12. QMOHI’s task times for these 2 groups were exceptionally high compared with the other groups.

The unresponsiveness of SHC websites for one of the universities in each of the 2 groups was detected to be the root cause behind this disparity. For most universities (179/200, 89.5%), QMOHI’s task time was less than a minute. For fewer universities (16/200,8.5%), the task time was under 2 minutes, and a handful of universities (3/200,1.5%) required 6 minutes or less. However, for 2 universities, the task times were 25 minutes and 32 minutes because of the unresponsiveness of the SHC websites.

We isolated the 2 outliers and compared the manual approach with QMOHI for the remaining 99% (198/200) universities. The average task time per university for the manual approach was 2.52 (SD 0.67) minutes and for QMOHI, it was 0.32 (SD 0.18) minutes. The QMOHI tool was more than 7 times faster than human annotators at the task of finding the first webpage with relevant information on the SHC website.

It is worth noting that this experiment studied the simplest of the metrics for both the tool and human annotators. Other quality metrics, such as readability and prevalence, which are more difficult for humans to assess, would likely increase the time for manual approach substantially. Empirical benchmarking of these task times will be a part of future work.

**Table 1:** Experiment 1—scalability. Aggregate group-level task times.

Method	Time (minutes)			
	Minimum time per group	Maximum time per group	Average time per group (SD)	Total time for all 20 groups
Annotator 1	11	40	23.95 (6.85)	479
Annotator 2	19	43	27.15 (6.85)	543
QMOHI^a^ tool	1	34	6.30 (9.62)	126

^a^QMOHI: Quantitative Measures of Online Health Information.

#### Method 2

Benchmarking revealed that QMOHI’s mean end-to-end runtime per university was 77.06 (SD 97.65) seconds for topic 1 (*pap smear;* number of hits=11) and 114.06 (SD 138.85) seconds for topic 2 (*all contraception;* number of hits=13). No relevant content was found in 9 sites for topic 1 and in 7 sites for topic 2.

The runtime of the tool was dominated by network-related tasks (ie, retrieving webpages). For topic 1, the network time accounted for 98.33% (75.78/77.06 seconds) of the total runtime. For topic 2, the network time accounted for 98.23% (112.03/114.06 seconds) of the total runtime. The tool’s processing time accounted only for 1.67% (1.29/77.06 seconds) and 1.77% (2.02/114.06 seconds) for topic 1 and topic 2, respectively.

The network times were less interesting to compare, as a human annotator would also experience similar latency retrieving the pages using their browser. However, the quality metric computation was consistently performed in a few seconds by the QMOHI tool, with only approximately 1 second slower performance for queries with 6-fold more keywords. This was in contrast to human annotation, which required a few minutes to read the content, and many more to perform the quality assessments manually. Overall, these results showed that the QMOHI tool is highly scalable and substantially more efficient than the manual approach.

### Experiment 2: Generalizability

Table 2 provides 6 quality metrics computed by QMOHI for information posted on the SHC website at SFSU for 4 distinct health topics (COVID, Cancer, LARC, and Condoms) and 2 closely related health topics (Hormonal IUD and Copper IUD). These results illustrated QMOHI tool’s versatility in terms of being applicable to any given topic as long as it was represented as a set of keywords. As such, the QMOHI tool could be used to study the information quality of a wide variety of topics.

Some of the observations from the metric values are as follows: navigation metric value of 0 for COVID aligns with the current trend on public health websites, which is to post a message related to COVID on the home page. The higher coverage of the *Condoms* topic compared with the other topics is expected as information dissemination on condoms is one of the focus areas for most SHC websites.

If the aforementioned results showcase the *breadth* ability of QMOHI, then the results in Table 2, group B demonstrate the *depth* ability of the tool. Table 2, group B provides information quality metrics for two closely related contraception methods: hormonal IUD and copper IUD (Paragard).

Overall, these results suggest that the QMOHI tool is capable of generating information quality metrics for any given topic. Users can customize the input keywords to the QMOHI tool for a particular topic of any granularity, making it a generic tool with broad applicability.

**Table 2:** Experiment 2—generalizability. Results showing the QMOHI (Quantitative Measures of Online Health Information) tool’s ability to compute information quality metrics for 4 diverse health topics (group A) and closely related health topics (group B).

Health topic			Readability (Flesch–Kincaid)					Navigation (number of clicks from home page)			Coverage (0–100)			Objectivity (0.0–1.0)			Polarity (-1.0 to 1.0)
			Reading ease score (0–100)		Grade level (K–12)												
**Group A: 4 diverse health topics**																	
	COVID	77.61		5.18		0			37.50			0.639			0.091		
	Cancer	76.06		5.81		1			11.11			0.542			0.182		
	LARC^a^	73.27		7.30		1			33.33			0.496			0.183		
	Condoms	75.42		6.12		1			100.00			0.373			0.111		
**Group B: 2 closely related health topics**																	
	Hormonal IUD^b^	73.19		7.98		1			42.86			0.486			0.161		
	Copper IUD	70.51		8.14		1			25.00			0.486			0.161		

^a^LARC: long-acting reversible contraceptives.

^b^IUD: intrauterine device.

### Experiment 3: Robustness

**Figure 3:**
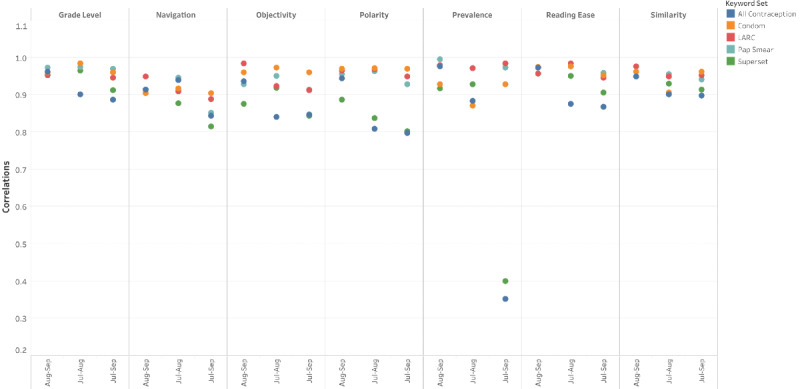
Experiment 3—robustness of QMOHI (Quantitative Measures of Online Health Information) tool. Pairwise correlation scores between metric values across the 3 monthly reruns of QMOHI (from July 2020 until September 2020) for the 5 health topics. LARC: long-acting reversible contraceptive.

Figure 3 illustrates the robustness of results in terms of the correlation between the metric values across the 3 reruns of QMOHI for the 5 health topics. For every metric, the pairwise correlation for the 3 time points (July, August, and September 2020) was computed.

As shown in Figure 3, most of the correlation values were close to 1, indicating high fidelity in the reproduction of the results across multiple time points. The absence of perfect correlation scores suggested that the metric values were time-varying because of the dynamic nature of information on the internet. Our analysis revealed two types of changes that had happened to the SHC websites between the tool’s reruns: (1) the content of the website had been updated and (2) the directory structure of the SHC website itself had changed. Table 3 provides a few examples of the second type of change.

**Table 3:** Experiment 3—robustness. Examples of QMOHI’s (Quantitative Measures of Online Health Information) ability to adapt to changing university student health center (SHC) website structure over 3 reruns.

University name	Old SHC website structure	New SHC website structure
University of Maryland Baltimore County	/studenthealth/services—hours/student-health-center/	/studenthealth/student-health-center
West Chester University of Pennsylvania	/_services/stu.inf/	/_services/studentHealthServices
Francis Marion University	/studenthealthservices/	/studentservices
Concord University	/student-life/node/35	/studenthealth
Coastal Carolina University	/services/studenthealth/	/health

## Discussion

### Principal Findings

In this study, we described a new open-source software tool, QMOHI, which has been designed and developed to quantify the quality of health information available on SHC websites. We then conducted an empirical evaluation of the QMOHI tool along three key performance metrics: scalability, generalizability, and robustness.

In our first evaluation, we showed that the navigation capabilities of QMOHI are at least seven times more efficient than the manual approach in determining web-based information quality. The runtime of the tool was dominated by network-related tasks. Once the relevant webpages are found, the processing times for computing the quality metrics are trivial. In contrast, human annotators would likely spend most of their time ascertaining information quality. In the second evaluation, we used a tool to retrieve quality metrics on broad and narrow health topics. We showed that once the user selects appropriate keywords, the tool can be adapted to any health topic, thereby establishing the generalizability and versatility of the tool. In the final evaluation, we redeployed QMOHI across 3 periods and showed that the tool is not vulnerable to typical structural changes to SHC websites, thereby allowing users to conduct longitudinal studies.

### Limitations

Currently one of the main limitations of QMOHI is its reliance on the keywords provided by users for the health topic of interest. The data gathered by the tool are entirely dependent on these keywords. The ability of the keywords to represent the health topic accurately and completely directly affects the accuracy of the information quality metrics provided by QMOHI. The keywords can also become a source of bias and thus influence the outcomes and conclusions drawn from studies in unexpected ways. One of the future directions of this work will explore automated keyword-generation approaches that require only the name of the health topic from the user and thus remove the dependence on user-provided keywords.

The data-gathering phase of QMOHI currently only collates textual information containing the keywords. This limits the information *visible* to the tool as relevant information is sometimes embedded in images and pdfs. To overcome this limitation, we plan to leverage recent advancements in computer vision to extract text from images and scanned documents.

The QMOHI project’s codebase can be downloaded and installed by following step-by-step instructions on the project webpage. In the future, we seek to take this a step further by providing a *plug-and-play* setup where minimal installation is needed. For this, we leverage the virtualization frameworks (eg, Docker) that are being increasingly adopted to lower the barriers for users with any background.

The applicability of QMOHI is currently restricted to the SHC websites of universities. This narrow focus was beneficial in terms of providing guardrails during the first cycle of project development. However, our goal is to lift this restriction and allow other web-based health information dissemination platforms to also use the quality assessment provided by QMOHI.

### Comparison With Previous Work

Health information quality assessment is an active field of research [9,11-13]. Nearly all existing approaches, including DARTS [11], study by Dobbins et al [12], DISCERN [9], and Lida [13], use surveys crafted by experts as the central tool for information quality assessment. These approaches can produce high-quality assessments, but are costly, time-consuming, and prone to human errors. QMOHI automates quality assessments by using natural language processing techniques in lieu of survey takers.

Table 4 shows how QMOHI fits in a sampling of the ecosystem of health information quality assessment tools. For a fair comparison, we combine QMOHI’s prevalence and coverage metrics as part of relevancy and QMOHI’s sentiment and polarity as part of reliability. DARTS, study by Dobbins et al [12], and DISCERN start with the assumption that the user has found a webpage of health information relevant to their interests. QMOHI and Lida start with the assumption that the user has (1) a specific health information need and (2) access to the internet. Lida does a superb job for assessing web usability, far more extensively than QMOHI (which assesses navigability only) and has a battery of automated tests to achieve those goals. Lida then relies on manual surveying to conduct information quality assessments. Many of these tools lack readability assessment and are used in conjunction with an external Flesch reading level analyzer [4-6]. QMOHI offers integrated Flesch-readability metrics. DARTS (Finland), study by Dobbins et al (Canada) [12], DISCERN (United Kingdom), and Lida (United Kingdom) were all developed outside of the United States; these tools rely on human survey takers, and are compatible with content in any language, provided that the survey is accessible to the survey takers. For example, DARTS specifically accommodates health care information in Finnish and English [44]. QMOHI focuses on university SHC websites in the United States. We believe that QMOHI offers a well-balanced and larger feature set than the existing tools. An empirical comparative analysis with tools such as AutoDISCERN [14,45] is part of future work.

**Table 4:** Comparison of information quality assessment tools.

Tool	Is fully automated	Is freely available	Assessments and metrics				
			Web usability	Readability	Reliability	Timeliness	Relevancy
DARTS^a^		✓			✓	✓	
Dobbins et al [12]		✓			✓	✓	✓
DISCERN		✓			✓	✓	✓
Lida			✓		✓	✓	✓
QMOHI^b^	✓	✓	✓	✓	✓	✓	✓

^a^Mnemonic for Date, Author, References, Type, Sponsors.

^b^QMOHI: Quantitative Measures of Online Health Information.

### Conclusions

This work introduced a new tool for public health research, QMOHI, that facilitates the scale monitoring of the quality of web-based information available on university SHC websites. QMOHI provides a suite of 8 metrics that quantify different aspects of information quality. Longitudinal studies that require periodic reexamination of the same information can be effectively facilitated by QMOHI. Such a tool can assist college health administrators in monitoring the recency and relevancy of the information provided on the SHC website. QMOHI can also be instrumental for centrally operated bodies, such as the ACHA, to help with the evaluation and standardization of health information on SHC websites of universities across the country. Overall, QMOHI is a powerful tool that can accelerate public health research based on web-based health information. QMOHI is an open-source project that is publicly available for nonprofit use [46].

## Appendix

Multimedia Appendix 1 Input keywords used with QMOHI (Quantitative Measures of Online Health Information) tool for the various health topics used in this study.

## Abbreviations

ACHA: American College Health Association
FKGL: Flesch–Kincaid Grade Level
FRE: Flesch Reading Ease
HEI: higher education institute
IUD: intrauterine device
LARC: long-acting reversible contraceptives
QMOHI: Quantitative Measures of Online Health Information
SFSU: San Francisco State University
SHC: student health center
