# Supplementary material for: Quantifying the Quality of Web-Based Health Information on Student Health Center Websites Using a Software Tool: Design and Development Study
Source: JMIR Form Res. 2022 Feb 2;6(2):e32360. doi: 10.2196/32360 (PMC8851325; doi:10.2196/32360)
Supplement: Multimedia Appendix 1 [file formative_v6i2e32360_app1.docx]

## Appendix

The input keywords used with QMOMI tool for the various health topics used in this study.

| Topic | Keywords used |
| --- | --- |
| COVID | “Corona”, “Coronavirus”, “Corona virus”, “Covid-19”, “Covid 19”, “COVID”, “SARS-CoV-2”, “2019nCoV” |
| Cancer | “Cancer”, “Biopsy”, “Chemoprevention”, “Chemotherapy”, “Leukemia”, “Lymphoma”, “Metastasis”, “Malignancy”, “Oncologist” |
| LARC | “IUD”, “Intrauterine device”, “IUI”, “Intrauterine implant”, “Contraceptive implant”, “Contraceptive shot”, “Contraceptive injection”, “Depo provera”, “Depo-provera” |
| Condom | “Condom”, “Condoms” |
| Hormonal IUD | “Hormonal IUD”, “Progesterone IUD”, “Progestin”, “Mirena”, “Skyla”, “Kyleena”, “Liletta” |
| Copper IUD | “Copper IUD”, “Non-Hormonal IUD”, “Nonhormonal IUD”, “Paragard” |
| Pap Smear | “pap smear”, “pap smears”, “papsmear”, “papsmears”, “pap test”, “pap tests” |
| All forms of contraception | “Birth Control”, “IUD”, “Progesterone IUD”, “Progestin”, “Hormonal IUD”, “Mirena”, “Skyla”, “Kyleena”, “Liletta”, “Copper IUD”, “Non-Hormonal IUD”, “Nonhormonal IUD”, “Paragard”, “Contraceptive implant”, “Nexplanon”, “Contraceptive injection”, “Control shot”, “Depo-Provera”, “Depo”, “emergency contraception”, “emergency contraceptives”, “morning after pill”, “Plan B”, “levonorgestrel”, “ella”, “ulipristal acetate”, “contraceptive pill”, “control pill”, “diaphragm”, “spermicide”, “contraceptive patch”, “control patch”, “vaginal ring”, “control ring”, “contraceptive ring”, “cervical cap” |
